# Anxiety Level During the Second Localized COVID-19 Pandemic Among Quarantined Infertile Women: A Cross-Sectional Survey in China

**DOI:** 10.3389/fpsyt.2021.647483

**Published:** 2021-07-22

**Authors:** Lian-Bao Cao, Qianjie Hao, Yan Liu, Qiang Sun, Bing Wu, Lili Chen, Lei Yan

**Affiliations:** ^1^School of Medicine, Cheeloo College of Medicine, Shandong University, Jinan, China; ^2^Department of Obstetrics and Gynecology, The Eighth People's Hospital of Xinjiang Uygur Autonomous Region, Urumqi, China; ^3^Department of Obstetrics and Gynecology, Zaozhuang Municipal Hospital, Zhaozhuang, China

**Keywords:** anxiety, COVID-19, mental health, quarantine, infertility

## Abstract

Infertility usually causes mental health problems for patients and unfavorable emotions such as anxiety and depression can have an adverse effect on women's normal pregnancy. We aimed to compare the anxiety level between infertile female patients in quarantined and non-quarantined areas during the second wave of COVID-19 epidemic. A total of 759 infertile women were included in this cross-sectional study conducted through an online survey. Anxiety was measured by the State-Trait Anxiety Inventory (STAI) tool. Participants were divided into the quarantined group (QG) and non-quarantined group (Non-QG). Independent sample *T*-test and chi-square test were performed to examine the difference between the two groups. There was no significant difference in the average STAI score of the two groups of infertile women, but responses to the emotional state showed that women in the QG had a higher tendency to be anxious. Participants in QG spent more time paying attention to the dynamics of the epidemic every day, and their sleep (*p* < 0.01) and mood conditions were worse (*p* < 0.01) than in the Non-QG. The family relationship of QG is more tense than non-QG. Through the research on the infertility treatment information of the overall research population, it is found the average STAI-State (STAI-S) (*p* = 0.031) score and STAI-Trait (STAI-T) (*p* = 0.005) score of women who were infertile for more than 3 years were significantly higher than those of women with <2 years. The STAI-T score of infertile women who underwent *in vitro* fertilization (IVF) was higher than that of non-IVF women (*p* = 0.007), but no significant difference was observed with the STAI-S score. To conclude, although the second wave of quarantine during COVID-19 epidemic did not significantly increase anxiety in infertile women, it did lead to an increase in other negative emotions and worse family relationships. Patients with long-term infertility treatment and those who have had IVF are more anxious subgroups.

## Introduction

The COVID-19 pandemic was first announced by the World Health Organization (WHO) in December, 2019 and is now spreading worldwide at an alarming rate. The pandemic has had a wide-ranging impact on people's lives and psychology (1, 2). In China, with the effective control of the COVID-19 epidemic, the first wave of the epidemic has been brought under control, however, it is still facing the impact of the second or third wave of some regional small-scale outbreaks. Beijing, Xinjiang, Qingdao, Guangzhou, and other important cities have all experienced second wave of the epidemic. The first wave of the epidemic made people realize the hazards of the novel coronavirus in terms of infectivity and pathogenicity, and the sudden arrival of the second wave accentuated people's fear (3).

Quarantine measures are adopted by many countries to combat the spread of COVID-19. Its side effects have gradually started attracting people's attention. Psychological distress such as anxiety and depression have shown widespread occurrence in the global pandemic of COVID-19 (1, 4). Our previous research has proved that in the second wave of the epidemic, quarantine can increase the anxiety levels of the population (3). Risk of getting infected by COVID-19 accompanied by quarantine and the national lockdown may lead to acute panic, anxiety, compulsive behavior, and other mental health problems (5). Among mental distress factors (anxiety, stress, and fear of COVID19), depressive symptoms play a vital role (6). Meta-analysis studies indicated that the quarantine does not have a uniformly adverse effect on mental health of population at risk, and the mental state of medical staff, patients with non-communicable chronic diseases, COVID-19 patients, and quarantined people are more likely to be affected (7, 8). Relationship between couples and the quality of sexual life are also affected by quarantine during COVID-19 epidemic (9, 10). Scholars began to notice these problems and called for effective preventive measures (11, 12).

The blockade due to the epidemic will inevitably affect the number of medical visits for some chronic diseases or clinical populations with regular follow-ups. During the delayed phase of the COVID-19 pandemic, pregnant women experience high levels of anxiety, and post-traumatic stress disorder (PTSD) symptoms (13). However, there are not many research reports on the psychological state of infertile female women during the pandemic (14, 15). The pressure of infertility is still high and higher than the pressure caused by the pandemic (16). For people with assisted reproductive treatments (ART), they need to go to the hospital for review on a regular basis to determine the next treatment plan. The sudden outbreak disrupted the normal medical treatment plan of infertility patients. Suspension of fertility treatment during the pandemic was taxing and as a result, negative emotional reactions were triggered (17, 18). For infertile patients, repeated treatment failures and long-term treatments can bring about psychological problems such as anxiety, especially for patients who have been identified as the infertility caused by female causes, they will experience more serious anxiety levels (19, 20). Previous research suggests going beyond psychiatric evaluation of infertile patients and focusing research efforts on the analysis of impaired quality of life in order to clinically address aspects related to infertility that can affect couples' well-being (21, 22). Relatively, bad mental states such as anxiety and depression will adversely affect the pregnancy outcome of infertile females (23–25). Infertility affects the relationship of both members of the couple, and women usually report worse adjustments to infertility and higher infertility related stress to man (26). Therefore, current study was conducted with the aim to explore whether quarantine will cause changes in the psychological state of infertile females.

Since July 15, 2020, Urumqi, Xinjiang has experienced the second wave of COVID-19 pandemic. Subsequently, the government adopted emergency isolation and blockade measures. The current study explores the anxiety levels among infertile women during the COVID-19-related quarantine. The aim of the current study is to compare the anxiety level between the quarantined and non-quarantined infertile women during the second wave of COVID-19.

## Methods

### Study Design

The cross-sectional study was conducted from August 20 to September 1, 2020 in the form of the “Questionnaire STAI” electronic questionnaire system (China Changsha Haoxing Information Technology Co., Ltd.). The research protocol was approved by the Ethics Committee of the Eighth People's Hospital of Xinjiang Uygur Autonomous Region. The informed consent form is displayed on the front page of the questionnaire and was accepted by the participants. All investigations are voluntary and anonymous.

Some items (such as age, marriage, pregnant, or not) are also used for invalid response and to ensure data quality. The inclusion criteria are: ① Chinese female citizens who live in mainland China and answer the set questions accurately; ② diagnosed of infertility; ③ information feedback can be provided through WeChat electronic questionnaire system. The exclusion criteria are: ① invalid responses; ② suffering from known mental illness; ③ already pregnant after treatment; ④ important data missing. According to the answers to the question about quarantine situation, the participants were divided into two groups: Quarantine Group (QG) and Non-Quarantine Group (Non-QG), which was also described in our previous paper (3).

### Questionnaire Design

The questionnaire includes aim, informed consent, general information, infertility information, and State-Trait Anxiety Inventory (STAI) component. The answers are expressed in ordered or unordered categorical variables. The content of the COVID-19 impact questionnaire has been introduced in detail in our previous research (3). The State-Trait Anxiety Scale has two subscales: State (STAI-S) and Trait (STAI-T) (27). STAI-S includes 20 items, which determine how an individual feels at a specific moment and under specific conditions. STAI-T has 20 items and usually determines how the participants feel, regardless of the situation. We defined STAI-S score higher than 53 or STAI-T score higher than 55 as suffering from severe anxiety disorder, which have been validated in the Chinese population before (28). The infertility information survey includes information as follows: ① History of pregnancy and child birth; ② Number of years of marriage; ③ Frequency of sex; ④ Have ever used assisted reproductive technology; ⑤ Current treatment methods; ⑥ Number of years of treatment.

### Statistical Analysis

The data were analyzed using SPSS 20 program (IBM Corporation, Illinois, USA). Independent samples *T*-test or one-way analysis of variance were used to compare the average of independent groups with a normal distribution. Bonferroni correction was performed when multiple independent statistical tests were being performed simultaneously. Chi-square test or Fisher's exact test was used to compare categorical variables. *P* < 0.05 is used for statistical significance. Quantitative variables are expressed as mean ± standard deviation (*SD*). Qualitative variables were expressed as numbers and percentages.

## Results

### Comparison of Anxiety Levels Between the Non-QG and QG

**Figure 1:**
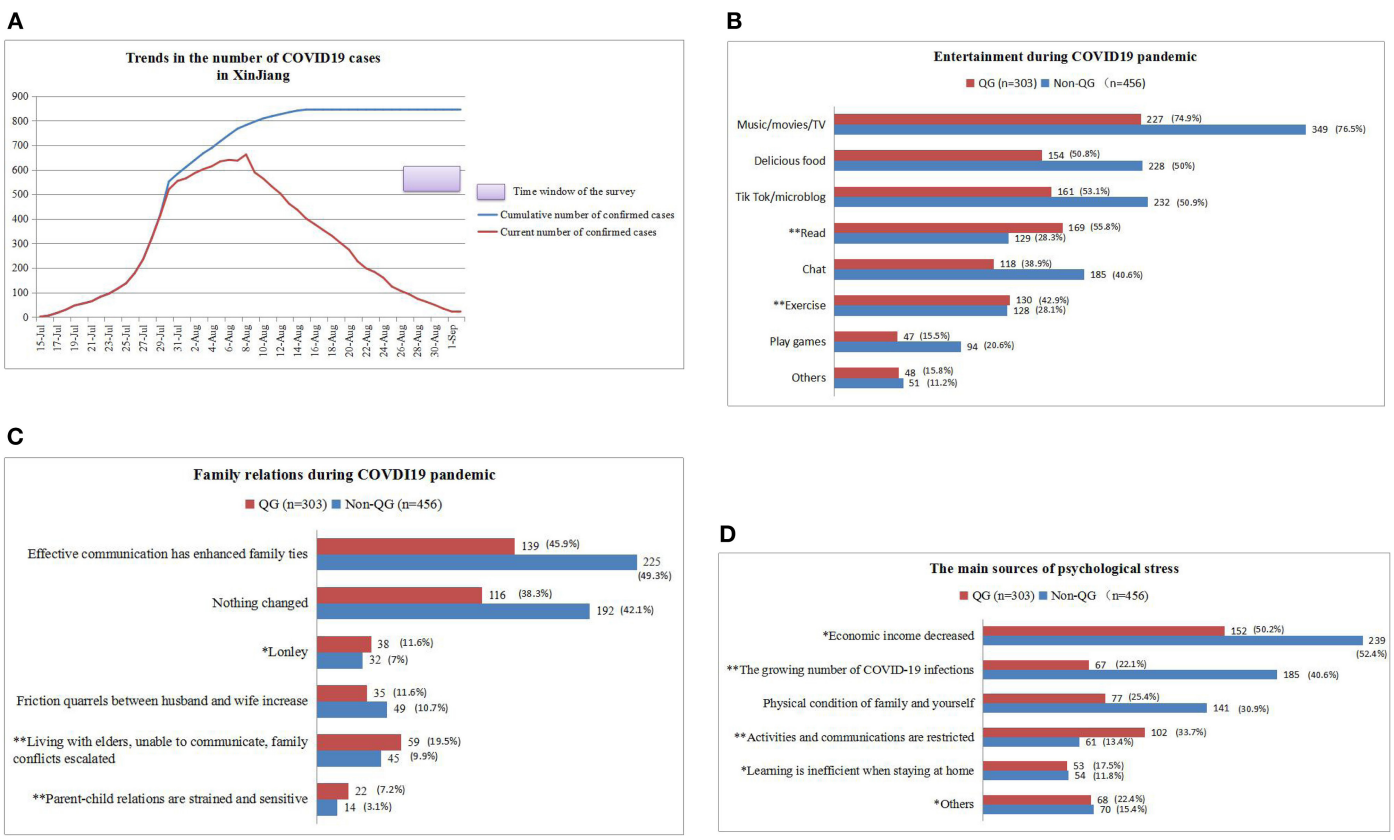
The entertainment methods, family relationships, and sources of psychological stress of infertile women in QG and non-QG during the epidemic period. **(A)** The trend of number of COVID-19 pneumonia patients and the survey window for this study. **(B)** Entertainment methods of the two groups of people during the COVID-19 epidemic. **(C)** Changes in the family relationship between the two groups during the COVID-19 epidemic. **(D)** Sources of psychological stress for the two groups during the COVID-19 epidemic. Quarantined group (QG), non-quarantined group (Non-QG), ^*^p < 0.05, ^**^p < 0.01.

A total of 1,943 participants from 27 provinces of China completed the questionnaire. 874 of the participants had been diagnosed with infertility. Among them, 115 patients were excluded based on exclusion criteria and 759 patients were finally included in the analysis, including 456 patients in non-QG and 303 patients in QG. In the non-QG group, 381 patients were diagnosed as primary infertility, with 229 patients with primary infertility in the QG groups (*P* = 0.339). The proportion of participants who had been infertile for more than 5 years did not differ statistically between the QG and non-QG groups except for the lack of related information in some patients (145/376 vs. 93/296, *P* = 0.055). Figure 1A showed the curve of number of COVID-19 pneumonia patients and the survey window. The questionnaire was delivered in the late stage of the second wave.

Table 1 showed the anxiety scores of the participants according to being quarantined or not. The mean STAI-S of all participants in the QG did not differ significantly compared to those in the Non-QG (40.7 ± 9.4 vs. 40.2 ± 9.1, *P* = 0.415), STAI-T score also had no significant difference (41.0 ± 9.2 vs. 41.6 ± 9.2, *P* = 0.421). There were also no statistical differences in the STAI-S and STAI-T scores of participants having different age, income levels, education levels, or health status.

**Table 1:** Comparison of STAI scores between the quarantined and non-quarantined groups.

**Characteristics**	**Sample size** **Non-QG**	**Sample size** **QG**	**STAI-S** **Non QG** **(Mean ± *SD*)**	**STAI-S** **QG** **(Mean ± *SD*)**	***F***	***df***	***P***	**STAI-T Non-QG** **(Mean ± *SD*)**	**STAI-T** **QG** **(Mean ± *SD*)**	***F***	***df***	***P***
**Total**	456	303	40.2 ± 9.1	40.7 ± 9.4	0.028	757	0.415	41.6 ± 9.2	41.0 ± 9.2	0.177	757	0.421
**Age(Y)**												
18–25	15	9	38.9 ± 7.5	43.6 ± 9.2	0.552	22	0.194	42.4 ± 7.9	43 ± 11.6	1.980	22	0.881
26–39	359	190	40.4 ± 9.3	41.3 ± 10.3	0.974	547	0.303	41.7 ± 9.4	41.6 ± 9.6	0.001	547	0.891
40–59	82	104	39.7 ± 8.6	39.6 ± 7.6	1.079	184	0.945	40.9 ± 8.6	39.8 ± 8.0	0.421	184	0.390
**Income**												
Low	221	126	41.6 ± 9.6	42.3 ± 9.9	0.008	345	0.279	42.3 ± 9.9	42.6 ± 8.9	0.539	345	0.518
Middle	122	134	39.8 ± 8.4	40.2 ± 9.7	0.623	254	0.763	41.2 ± 8.5	40.9 ± 9.6	0.436	254	0.791
High	113	43	39.8 ± 8.8	39.7 ± 8.9	0.158	154	0.925	40.5 ± 8.6	39.7 ± 8.6	0.313	154	0.578
**Education**												
High school or below	187	73	40.5 ± 9.5	42.0 ± 8.1	2.364	258	0.233	42.0 ± 9.7	42.0 ± 8.0	1.434	258	0.977
College or Bachelor	224	212	40.2 ± 9.2	40.3 ± 9.8	0.398	434	0.908	41.6 ± 9.1	40.8 ± 9.4	0.005	434	0.365
Master or Doctor	45	18	38.8 ± 7.3	40.9 ± 10.0	0.962	61	0.343	39.8 ± 7.6	39.7 ± 10.9	1.996	61	0.974
**Occupation**												
Employees of institutions or government	137	170	40.0 ± 8.0	40.6 ± 10.0	3.249	305	0.566	41.7 ± 8.5	40.8 ± 9.9	0.609	305	0.419
Other employees or retired or students	319	133	40.3 ± 9.6	40.9 ± 8.8	1.340	450	0.487	41.5 ± 9.6	41.3 ± 8.2	1.652	450	0.808
**Health status**												
Very healthy	169	49	37.9 ± 8.9	39.4 ± 8.7	0.030	216	0.299	39.4 ± 9.0	37.3 ± 7.8	0.781	216	0.125
Relatively good	200	187	40.9 ± 8.3	39.9 ± 9.1	0.461	385	0.237	41.9 ± 8.7	40.9 ± 9.0	0.074	385	0.283
Moderate or bad	87	67	42.9 ± 10.4	44.1 ± 10.2	0.003	152	0.457	44.9 ± 9.9	44.0 ± 9.5	0.002	152	0.558

*STAI-S, State-Trait Anxiety Inventory-State; STAI-T, State-Trait Anxiety Inventory-Trait; QG, quarantined group; Non-QG, non-quarantined group; Y, year. P-values < 0.05 are in bold typeface*.

In order to further compare the severity of anxiety between the two groups, we analyzed its presence according to different factors (Table 2). Similarly, between the QG group and the non-QG group, there was no significant difference in the proportion of severe anxiety measured by STAI-S (7.9 vs. 7.0%, *P* = 0.641), and the STAI-T measurement results also showed no significant difference (7.6 vs. 7.9%, *P* = 0.878). A detailed analysis of the subgroups also showed that the remaining comparisons were not significantly different.

**Table 2:** Comparison of rates of severe anxiety between the quarantined and non-quarantined groups.

**Characteristics**	**Rate in Non-QG (STAI-S, %)**	**Rate in QG (STAI-S, %)**	***χ*****^2^**	***P***	**OR(95%CI)**	**Rate in Non-QG (STAI-T, %)**	**Rate in QG (STAI-T, %)**	***χ*****^2^**	***P***	**OR (95%CI)**
**Total**	(32/456)	(24/303)	0.217	0.641	0.877(0.506–1.521)	(36/456)	(23/303)	0.023	0.878	1.043 (0.605–1.799)
Sum	7.4 (56/759)^†^					7.8 (59/759)^†^				
**Age (Y)**										
18–25	6.7 (1/15)	11.1 (1/9)	0.145	1.000	0.571 (0.031–10.435)	13.4 (2/15)	11.1 (1/9)	0.025	1.000	1.231 (0.095–15.872)
26–39	7.2 (26/359)	10.5 (20/190)	1.745	0.186	0.664 (0.360–1.223)	8.1 (29/359)	9.5 (18/190)	0.309	0.578	0.840 (0.453–1.555)
40–59	6.1 (5/82)	2.9 (3/104)	1.150	0.304	2.186 (0.507–9.430)	6.1 (5/82)	3.8 (4/104)	0.505	0.511	1.632 (0.422–6.249)
**Income**										
Low	8.1 (18/221)	10.3 (13/126)	0.466	0.495	0.771 (0.364–1.631)	10.0 (22/221)	7.1 (9/126)	0.780	0.377	1.437 (0.640–3.226)
Middle	6.6 (8/122)	6.0 (8/134)	0.038	0.846	1.105 (0.402–3.041)	6.6 (8/122)	8.2 (11/134)	0.254	0.615	0.785 (0.305–2.020)
High	5.3 (6/113)	7.0 (3/43)	0.159	0.707	0.748 (0.178–3.133)	5.3 (6/113)	7.0 (3/43)	0.159	0.707	0.748 (0.178–3.133)
**Education**										
High school or below	6.4 (12/187)	8.2 (6/73)	0.265	0.607	0.766 (0.276–2.123)	9.1 (17/187)	4.1 (3/73)	1.835	0.176	2.333 (0.663–8.214)
College or Bachelor	(19/224)	(17/212)	0.031	0.861	1.063 (0.537–2.105)	(18/224)	(18/212)	0.030	0.863	0.942 (0.476–1.863)
Master or Doctor	2.2 (1/45)	5.6 (1/18)	0.465	0.493	0.386 (0.023–6.532)	2.2 (1/45)	11.1 (2/18)	2.240	0.194	0.182 (0.015–2.145)
**Occupation**										
Employees of institutions or government	5.1 (7/137)	7.6 (13/170)	0.802	0.370	0.650 (0.252–1.678)	8.0 (11/137)	8.8 (15/170)	0.062	0.804	0.902 (0.400–2.033)
Other employees or retired or students	7.8 (25/319)	8.3 (11/133)	0.024	0.877	0.943 (0.450–1.976)	7.8 (25/319)	6.0 (8/133)	0.460	0.497	1.329 (0.583–3.026)
**Health status**										
Very healthy	4.7 (8/169)	4.1 (2/49)	0.037	1.000	1.168 (0.240–5.687)	6.5 (11/169)	2.0 (1/49)	1.458	0.307	3.342 (0.421–26.548)
Relatively good	6.5 (13/200)	6.4 (12/187)	0.001	0.974	1.014 (0.450–2.282)	6.5 (13/200)	7.0 (13/187)	0.031	0.859	0.930 (0.420–2.063)
Moderate or bad	12.6 (11/87)	14.9 (10/67)	0.167	0.683	0.825 (0.328–2.076)	13.8 (12/87)	13.4 (9/67)	0.004	0.949	1.031 (0.407–2.613)

*STAI-S, State-Trait Anxiety Inventory-State; STAI-T, State-Trait Anxiety Inventory-Trait; QG, quarantined group; Non-QG, non-quarantined group; Y, year; NA, not applicable; CI, confidence interval; OR, odds ratio. P-values < 0.05 are in bold typeface*.

† *Sum of the two groups*.

**Figure 2:**
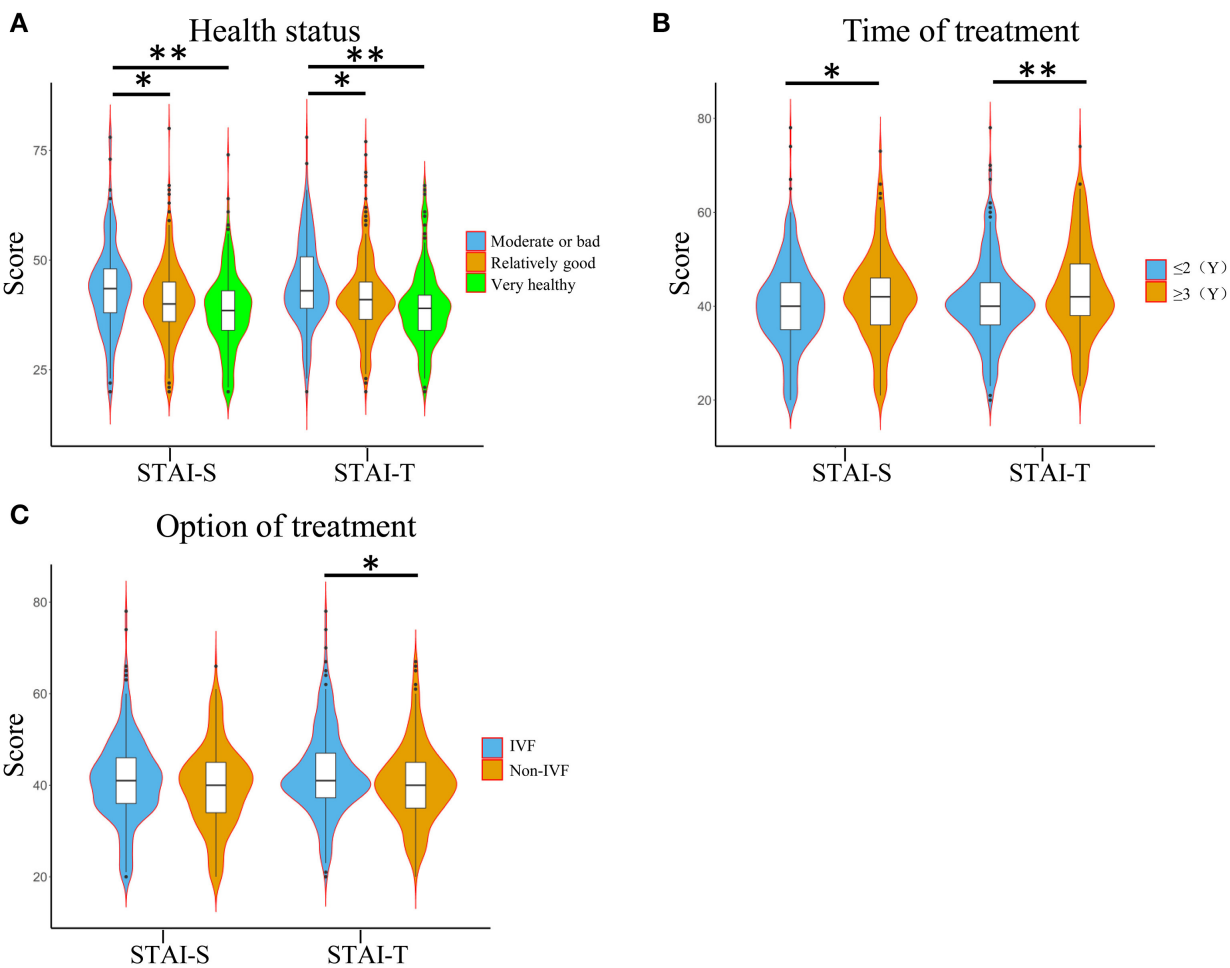
Anxiety score of subgroup level in the included population (*n* = 759). **(A)** The STAI-S and STAI-T score levels in subgroups of different health status. **(B)** The score levels of STAI-S and STAI-T in the subgroups of different treatment methods. **(C)** The score levels of STAI-S and STAI-T in the subgroups of different treatment time. STAI-S, State-Trait Anxiety Inventory-State; STAI-T, State-Trait Anxiety Inventory-Trait; IVF, *in vitro* fertilization. ^*^P < 0.05. ^**^P < 0.01.

The analysis of the overall research population found that there is no significant difference in the STAI scores of different age, income, education, and occupation subgroups (Supplementary Table 1). The STAI scores of different health status subgroups (moderate or bad, relatively good, and very healthy) were significantly different, and the anxiety degree of normal health status was significantly less than that of poor health status (*p* < 0.001, Figure 2A). Besides, we also found that the average STAI-S (41.8 ± 9.4 vs. 40.0 ± 9.1, *p* = 0.031) score and STAI-T (43.3 ± 9.9 vs. 40.9 ± 8.8, *p* = 0.005) score of women with time to treatment of more than 3 years were significantly higher than those for patients with time of treatment for < 2 years. Compared to patients with non-IVF, the STAI-T score of IVF patients was significant higher (42.7 ± 9.4 vs. 40.4 ± 8.5, *p* = 0.007), but the STAI-S score was not significantly different (41.0 ± 9.2 vs. 39.6 ± 9.0, *p* = 0.096) (Figures 2B,C).

### Other Psychological Effects of the Second COVID-19 Confinement

Table 3 summarizes other psychological findings of study participants related to the COVID-19 pandemic. The percentage of responses between the two groups also showed a similar trend. There was no significant difference between the two groups in their attitudes toward the COVID-19 pandemic and the feeling of relaxation after the pandemic was reduced. However, QG participants spent more time paying attention to the news about COVID-19 (*P* < 0.001), were more afraid or confused about the news about COVID-19 (*P* < 0.001), and also mood became worse and irritable (*P* < 0.001). The quality of sleep in the QG group was significantly worse (*P* < 0.001). The proportion of participants who felt lonely and depressed in QG was significantly higher from that in non-QG (*P* < 0.001). Our questionnaire also showed that the proportion of female patients in quarantine areas who need psychological counseling is higher than that in non-quarantine areas (16.8 vs. 10.1%, *P* = 0.006).

**Table 3:** Summary of the responses on the emotional and somatic state.

**Questions about feelings on COVID19 pandemic (showed in percentage and grayscale)**	**Non-QG (*****n*** **%) Total = 456**			**QG (*****n*** **%) Total = 303**		
	Strong			Strong		
	Low			Low		
Attitude toward COVID19 pandemic	3.7	71.3	25	3	72.6	24.4
Time for concerning news COVID19^**^	26.5	32.5	41	11.9	21.1	67
Scared or confused about COVID19 news^**^	65.8	32.5	1.8	59	35.3	5.7
Quality of sleep becomes poor^**^	88.1	11.2	0.7	60.4	31.7	7.9
Mood getting worse and irascible^**^	81.8	15.8	2.4	61	31.4	7.6
Feel lonely and depressed because lack of social activities^**^	82.5	15.4	2.2	64.4	28.1	7.6
Still not relaxed after the pandemic alleviated^*^	11	74.1	14.9	11.9	66	22.1

*QG, quarantined group; Non-QG, non-quarantined group*.

* *P < 0.05*.

** *P < 0.01*.

*The shade of the color represents the proportion of it. The darker the blue, the higher the proportion*.

Compared with non-QG, women in QG prefer to read books (28.3 vs. 55.8%, *p* < 0.01) and exercise (28.1 vs. 42.9%, *p* < 0.01) for entertainment (Figure 1B). The family relationship of QG is more tense than non-QG: the proportion of women who feel lonely in the family was higher in the QG group (11.6 vs. 7%, *p* < 0.05); the proportion of women who have escalated family conflicts was higher in the QG group (19.5 vs. 9.9%, *p* < 0.01); the relationship between parents and children was more strained and sensitive (7.2 vs. 3.1%, *p* < 0.01) (Figure 1C). The top three sources of psychological stress among non-QG: ① Economic income decreased; ② the growing number of COVID-19 infections; ③ Physical condition of family and yourself. The top three sources of psychological stress among QG: ① Economic income decreased; ② Activities and communications are restricted; ③ Physical condition of family and the patient (Figure 1D).

## Discussion

This study focused on the impact of quarantine on the anxiety level of infertile female during the second wave of COVID-19 outbreak in Xinjiang. Result from our study indicated that, quarantine measures did not significantly affect the anxiety level of the two groups (QG vs. non-QG). But we also found that infertile women in quarantine group pay more attention to the dynamics of the epidemic than infertile women in non-quarantine group, and their mood, tension, and sleep were all affected to varying degrees. It indicated that the quarantine may increase the psychological pressure of infertile women. There is growing evidence that most infertile women postpone examination and treatment during the pandemic, which may have a negative impact on their lives (17, 29). It has previously been reported that among infertile women whose ART cycle has been postponed due to the pandemic, women older than 35 years have higher levels of anxiety (30). Despite the COVID-19 pandemic, infertility is still among the top stressors, which can be comparable to the pressure caused by the epidemic itself (16). In April 2020, Ben-Kimhy et al. found in a study in Israel that despite the possible risk of infection and influence to embryos, most of infertile patients still expect to resume infertility treatment (31). Indeed, most infertile women are relatively young and healthy, and COVID-19 itself may not be as stressful as infertility. Regardless of whether it is a quarantine area or a non-quarantine area, economic pressure ranks first among infertile women. Interestingly, compared with COVID-19 itself, infertile women in the QG were more concerned about the restrictions on activities brought by quarantine.

Fertility is an important life decision for women, and the excessive moratorium caused by infertility will undoubtedly bring psychological pressure to women (32). The mental state of infertile patients has been reported by many researchers (33, 34). Studies have shown that coping with infertility is related to the periodic increase in psychological symptoms of distress, depression, and anxiety (35, 36). Women bear greater anxiety during treatment than their partners (32). Our study shows that a longer period of infertility did cause a higher level of anxiety than shorter period of infertility in women. Previous study indicated that women who have suffered from infertility for 2–3 years have the highest level of depression (37). Although IVF has brought new hope for infertile couples, the low success rate of IVF also brought a heavy burden (38). Compared with non-IVF patients, IVF patients had higher anxiety score and this may be as a result of IVF being the last resort for fertility treatment and can be more expensive than non-IVF. Women who received IVF treatment experienced increased levels of anxiety and depression on the day of oocytes retrieval, during embryo transfer and during the 2 weeks waiting for the embryo (39–41). People who failed to conceive a child through IVF have significantly higher levels of anxiety than those who attempt successfully (42). Although whether the increase or decrease in anxiety level will affect the results of IVF is still ambiguous (43). We still hope that the mental health of infertile women can receive attention, especially during the epidemic.

The psychological state of the quarantined people has also been reported by researchers from different countries (44–46). Fear of COVID-19 and mandatory quarantine measures have had a great impact on people's psychological state (45, 47). We found higher proportion of infertile women in the quarantine group for psychological counseling than that in the non-quarantine group. In Italy, the COVID-19 pandemic itself and the recommendation to stop the ART program have been shown to create higher levels of distress among infertile couples (14). The situation of infertile patients feeling helpless after discontinuation of treatment is related to higher distress, which is also reported by Israeli scholars (31).

The impact of the second wave of the epidemic on people is different from that of the first wave. The quarantine experience in the first wave of epidemic has eased people's anxiety to a certain level. We found that there are more conflicts in women's family relationships in the quarantine group during the second wave. Quarantine measures have brought many factors (isolation, incurable, infection) that may increase anxiety, however, the companionship and communication of family members, and a variety of entertainment methods may help relieve anxiety. Intimacy, increased communication, and commitment can effectively alleviate the tension between husband and wife (26). From another perspective, the performance of the Chinese government in the first wave of the epidemic produced a certain degree of confidence among the people for the government's response to the emergency situation. Compared with the lack of understanding of the unknown new virus during the first wave of epidemic, in the face of the second wave of the epidemic, people's psychological state also underwent a process of adaptation. When infertile patients cannot visit hospitals for treatment, it is recommended that clinicians provide patients with psychological and lifestyle guidance through online forms. The role of social assistance cannot be ignored. The mutual communication between family and friends, and a good relationship between husband and wife are all conducive to the mental health of infertile women (31).

There are several limitations in our study; first, of which lies in the cross-sectional nature of data, without a baseline assessment of anxiety before the pandemic, or at least during the first months of the pandemic. Second, we used the mobile WeChat questionnaire to conduct surveys, so the women who felt good may have a higher response rate. Third, the number of confirmed infections in Xinjiang during the window period of our investigation had shown a clear downward trend, and people's anxiety at that time might have been relieved. Fourth, there are some disadvantages based on the cross-sectional study itself. For example, it may include data on confounding factors and other variables that affect the assumed causality. Fifth, we have no information about the cause of infertility and the number of attempts. In addition, the self-reported diagnosis may also have some deviations in data collection.

In the future, research across different regions of China and research including the mental state of male infertile patients will help to further expand our understanding of the impact that quarantine measures on the mental state of infertile patients.

## Conclusion

Our research found that there was no significant difference in the anxiety level of infertile females in Xinjiang quarantine area under the second wave of epidemics compared with patients in non-quarantine areas. However, quarantine could still lead to an increase in negative emotions and deterioration of family relationships; infertile patients of quarantine are people who need more psychological counseling and care. Patients with long-term infertility treatment and those who need to do IVF are more anxious.

## Data Availability Statement

The raw data supporting the conclusions of this article will be made available by the authors, without undue reservation.

## Ethics Statement

The studies involving human participants were reviewed and approved by Ethics Committee of the Eighth People's Hospital of Xinjiang Uygur Autonomous Region. Written informed consent for participation was not required for this study in accordance with the national legislation and the institutional requirements.

## Author Contributions

L-BC: original draft preparation. QH, YL, QS, and BW: data collection. L-BC: writing—reviewing and editing. LY: study design, statistical analysis, and revise draft articles. All authors contributed, reviewed, and approved the final manuscript.

## Conflict of Interest

The authors declare that the research was conducted in the absence of any commercial or financial relationships that could be construed as a potential conflict of interest.

## References

[1] Brooks SK Webster RK Smith LE Woodland L Wessely S Greenberg N. The psychological impact of quarantine and how to reduce it: rapid review of the evidence. Lancet. (2020) 395:912–20. 10.1016/S0140-6736(20)30460-8 32112714 PMC7158942

[2] Dubey S Biswas P Ghosh R Chatterjee S Dubey MJ Chatterjee S. Psychosocial impact of COVID-19. Diabetes Metab Syndr. (2020) 14:779–88. 10.1016/j.dsx.2020.05.035 32526627 PMC7255207

[3] Chen L Zhao H Razin D Song T Wu Y Ma X. Anxiety levels during a second local COVID-19 pandemic breakout among quarantined people: a cross sectional survey in China. J Psychiatr Res. (2021) 135:37–46. 10.1016/j.jpsychires.2020.12.067 33445059 PMC7783475

[4] Necho M Tsehay M Birkie M Biset G Tadesse E. Prevalence of anxiety, depression, and psychological distress among the general population during the COVID-19 pandemic: a systematic review and meta-analysis. Int J Soc Psychiatry. (2021) 2021:207640211003121. 10.1177/00207640211003121 33794717

[5] Marel C Mills KL Teesson M. Substance use, mental disorders and COVID-19: a volatile mix. Curr Opin Psychiatry. (2021) 34:351–6. 10.1097/YCO.0000000000000707 33741762 PMC8183242

[6] Di Blasi M Gullo S Mancinelli E Freda MF Esposito G Gelo OCG. Psychological distress associated with the COVID-19 lockdown: a two-wave network analysis. J Affect Disord. (2021) 284:18–26. 10.1016/j.jad.2021.02.016 33582428 PMC8771473

[7] Prati G Mancini AD. The psychological impact of COVID-19 pandemic lockdowns: a review and meta-analysis of longitudinal studies and natural experiments. Psychol Med. (2021) 51:201–11. 10.1017/S0033291721000015 33436130 PMC7844215

[8] Wu T Jia X Shi H Niu J Yin X Xie J. Prevalence of mental health problems during the COVID-19 pandemic: a systematic review and meta-analysis. J Affect Disord. (2021) 281:91–8. 10.1016/j.jad.2020.11.117 33310451 PMC7710473

[9] Luetke M Hensel D Herbenick D Rosenberg M. Romantic relationship conflict due to the COVID-19 pandemic and changes in intimate and sexual behaviors in a nationally representative sample of american adults. J Sex Marital Ther. (2020) 46:747–62. 10.1080/0092623X.2020.1810185 32878584

[10] Panzeri M Ferrucci R Cozza A Fontanesi L. Changes in sexuality and quality of couple relationship during the COVID-19 lockdown. Front Psychol. (2020) 11:565823. 10.3389/fpsyg.2020.565823 33132969 PMC7550458

[11] McGinty EE Presskreischer R Han H Barry CL. Psychological distress and loneliness reported by US adults in 2018 and April (2020). JAMA. (2020) 324:93–4. 10.1001/jama.2020.9740 32492088 PMC7270868

[12] Qiu J Shen B Zhao M Wang Z Xie B Xu Y. A nationwide survey of psychological distress among Chinese people in the COVID-19 epidemic: implications and policy recommendations. Gen Psychiatr. (2020) 33:e100213. 10.1136/gpsych-2020-100213 32215365 PMC7061893

[13] Hocaoglu M Ayaz R Gunay T Akin E Turgut A Karateke A. Anxiety and post-traumatic stress disorder symptoms in pregnant women during the COVID-19 pandemic's delay phase. Psychiatr Danub. (2020) 32:521–6. 10.24869/psyd.2020.521 33370762

[14] Esposito V Rania E Lico D Pedri S Fiorenza A Strati MF. Influence of COVID-19 pandemic on the psychological status of infertile couples. Eur J Obstet Gynecol Reprod Biol. (2020) 253:148–53. 10.1016/j.ejogrb.2020.08.025 32866858 PMC7443353

[15] Gordon JL Balsom AA. The psychological impact of fertility treatment suspensions during the COVID-19 pandemic. PLoS ONE. (2020) 15:e0239253. 10.1371/journal.pone.0239253 32946479 PMC7500693

[16] Vaughan DA Shah JS Penzias AS Domar AD Toth TL. Infertility remains a top stressor despite the COVID-19 pandemic. Reprod Biomed Online. (2020) 41:425–7. 10.1016/j.rbmo.2020.05.015 32600945 PMC7274108

[17] Boivin J Harrison C Mathur R Burns G Pericleous-Smith A Gameiro S. Patient experiences of fertility clinic closure during the COVID-19 pandemic: appraisals, coping and emotions. Hum Reprod. (2020) 35:2556–66. 10.1093/humrep/deaa218 32761248 PMC7454659

[18] Marom Haham L Youngster M Kuperman Shani A Yee S Ben-Kimhy R Medina-Artom TR. Suspension of fertility treatment during the COVID-19 pandemic: views, emotional reactions and psychological distress among women undergoing fertility treatment. Reprod Biomed Online. (2021) 42:849–58. 10.1016/j.rbmo.2021.01.007 33558171 PMC7816616

[19] Gdanska P Drozdowicz-Jastrzebska E Grzechocinska B Radziwon-Zaleska M Wegrzyn P Wielgos M. Anxiety and depression in women undergoing infertility treatment. Ginekol Pol. (2017) 88:109–12. 10.5603/GP.a2017.0019 28326521

[20] Massarotti C Gentile G Ferreccio C Scaruffi P Remorgida V Anserini P. Impact of infertility and infertility treatments on quality of life and levels of anxiety and depression in women undergoing *in vitro* fertilization. Gynecol Endocrinol. (2019) 35:485–9. 10.1080/09513590.2018.1540575 30612477

[21] Aarts JW van Empel IW Boivin J Nelen WL Kremer JA Verhaak CM. Relationship between quality of life and distress in infertility: a validation study of the Dutch FertiQoL. Hum Reprod. (2011) 26:1112–8. 10.1093/humrep/der051 21372046

[22] Boivin J Takefman J Braverman A. The fertility quality of life (FertiQoL) tool: development and general psychometric properties. Hum Reprod. (2011) 26:2084–91. 10.1093/humrep/der171 21665875 PMC3137391

[23] Klonoff-Cohen H Chu E Natarajan L Sieber W. A prospective study of stress among women undergoing *in vitro* fertilization or gamete intrafallopian transfer. Fertil Steril. (2001) 76:675–87. 10.1016/S0015-0282(01)02008-8 11591398

[24] Terzioglu F Turk R Yucel C Dilbaz S Cinar O Karahalil B. The effect of anxiety and depression scores of couples who underwent assisted reproductive techniques on the pregnancy outcomes. Afr Health Sci. (2016) 16:441–50. 10.4314/ahs.v16i2.12 27605959 PMC4994561

[25] Xu H Ouyang N Li R Tuo P Mai M Wang W. The effects of anxiety and depression on *in vitro* fertilisation outcomes of infertile Chinese women. Psychol Health Med. (2017) 22:37–43. 10.1080/13548506.2016.1218031 27686881

[26] Donarelli Z Lo Coco G Gullo S Salerno L Marino A Sammartano F. The Fertility Quality of Life Questionnaire (FertiQoL) Relational subscale: psychometric properties and discriminant validity across gender. Hum Reprod. (2016) 31:2061–71. 10.1093/humrep/dew168 27343271

[27] Spielberger CD Gorsuch R Lushene R. STAI Manual for the State-Trait Anxiety Inventory. Palo Alto CA: Consulting Psychologists Press (1970).

[28] Wang X Wang X Ma H. Handbook of Mental Health Rating Scale (revised edition). Beijing Chin J Ment Health Suppl Edn. (1999). 1999:205–9.

[29] Barra F La Rosa VL Vitale SG Commodari E Altieri M Scala C. Psychological status of infertile patients who had *in vitro* fertilization treatment interrupted or postponed due to COVID-19 pandemic: a cross-sectional study. J Psychosom Obstet Gynaecol. (2020) 2020:1–8. 10.1080/0167482X.2020.1853095 33252292

[30] Tokgoz VY Kaya Y Tekin AB. The level of anxiety in infertile women whose ART cycles are postponed due to the COVID-19 outbreak. J Psychosom Obstet Gynaecol. (2020) 2020:1–8. 10.1080/0167482X.2020.1806819 32812477

[31] Ben-Kimhy R Youngster M Medina-Artom TR Avraham S Gat I Haham LM. Fertility patients under COVID-19: attitudes, perceptions, and psychological reactions. Hum Reprod. (2020) 35:2774–83. 10.1093/humrep/deaa248 32877507 PMC7499650

[32] Malina A Blaszkiewicz A Owczarz U. Psychosocial aspects of infertility and its treatment. Ginekol Pol. (2016) 87:527–31. 10.5603/GP.2016.0038 27504947

[33] Frederiksen Y Farver-Vestergaard I Skovgard NG Ingerslev HJ Zachariae R. Efficacy of psychosocial interventions for psychological and pregnancy outcomes in infertile women and men: a systematic review and meta-analysis. BMJ Open. (2015) 5:e006592. 10.1136/bmjopen-2014-006592 25631310 PMC4316425

[34] Ying L Wu LH Loke AY. The effects of psychosocial interventions on the mental health, pregnancy rates, and marital function of infertile couples undergoing *in vitro* fertilization: a systematic review. J Assist Reprod Genet. (2016) 33:689–701. 10.1007/s10815-016-0690-8 26979745 PMC4889475

[35] Eugster A Vingerhoets AJ. Psychological aspects of *in vitro* fertilization: a review. Soc Sci Med. (1999) 48:575–89. 10.1016/S0277-9536(98)00386-4 10080360

[36] Li J Liu B Li M. Coping with infertility: a transcultural perspective. Curr Opin Psychiatry. (2014) 27:320–5. 10.1097/YCO.0000000000000091 25023887

[37] Domar AD Broome A Zuttermeister PC Seibel M Friedman R. The prevalence and predictability of depression in infertile women. Fertil Steril. (1992) 58:1158–63. 10.1016/S0015-0282(16)55562-9 1459266

[38] Ishihara O Adamson GD Dyer S de Mouzon J Nygren KG Sullivan EA. International committee for monitoring assisted reproductive technologies: world report on assisted reproductive technologies. (2007). Fertil Steril. (2015). 103:402.e11–13.e11. 10.1016/j.fertnstert.2014.11.004 25516078

[39] Verhaak CM Smeenk JM Evers AW Kremer JA Kraaimaat FW Braat DD. Women's emotional adjustment to IVF: a systematic review of 25 years of research. Hum Reprod Update. (2007) 13:27–36. 10.1093/humupd/dml040 16940360

[40] Wang K Li J Zhang JX Zhang L Yu J Jiang P. Psychological characteristics and marital quality of infertile women registered for *in vitro* fertilization-intracytoplasmic sperm injection in China. Fertil Steril. (2007) 87:792–8. 10.1016/j.fertnstert.2006.07.1534 17222834

[41] Yong P Martin C Thong J. A comparison of psychological functioning in women at different stages of *in vitro* fertilization treatment using the mean affect adjective check list. J Assist Reprod Genet. (2000) 17:553–6. 10.1023/A:1026429712794 11209535 PMC3455452

[42] Csemiczky G Landgren BM Collins A. The influence of stress and state anxiety on the outcome of IVF-treatment: psychological and endocrinological assessment of Swedish women entering IVF-treatment. Acta Obstet Gynecol Scand. (2000) 79:113–8. 10.1034/j.1600-0412.2000.079002113.x 10696958

[43] Zaig I Azem F Schreiber S Gottlieb-Litvin Y Meiboom H Bloch M. Women's psychological profile and psychiatric diagnoses and the outcome of *in vitro* fertilization: is there an association? Arch Womens Ment Health. (2012) 15:353–9. 10.1007/s00737-012-0293-z 22767032

[44] Lopez-Bueno R Calatayud J Ezzatvar Y Casajus JA Smith L Andersen LL. Association between current physical activity and current perceived anxiety and mood in the initial phase of COVID-19 confinement. Front Psychiatry. (2020) 11:729. 10.3389/fpsyt.2020.00729 32793013 PMC7390883

[45] Schuch FB Bulzing RA Meyer J Vancampfort D Firth J Stubbs B. Associations of moderate to vigorous physical activity and sedentary behavior with depressive and anxiety symptoms in self-isolating people during the COVID-19 pandemic: a cross-sectional survey in Brazil. Psychiatry Res. (2020) 292:113339. 10.1016/j.psychres.2020.113339 32745795 PMC7384423

[46] Zhang C Yang L Liu S Ma S Wang Y Cai Z. Survey of insomnia and related social psychological factors among medical staff involved in the 2019. Novel coronavirus disease outbreak. Front Psychiatry. (2020) 11:306. 10.3389/fpsyt.2020.00306 32346373 PMC7171048

[47] Mosheva M Hertz-Palmor N Dorman Ilan S Matalon N Pessach IM Afek A. Anxiety, pandemic-related stress and resilience among physicians during the COVID-19 pandemic. Depress Anxiety. (2020) 37:965–71. 10.1002/da.23085 32789945 PMC7436709

