# Supplementary material for: Anxiety Level During the Second Localized COVID-19 Pandemic Among Quarantined Infertile Women: A Cross-Sectional Survey in China
Source: Front Psychiatry. 2021 Jul 22;12:647483. doi: 10.3389/fpsyt.2021.647483 (PMC8339465; doi:10.3389/fpsyt.2021.647483)
Supplement: Supplementary file 1 [file Table_1.DOCX]

| Characteristics | Sample size | STAI-S  (Mean±SD) | F | df | P | STAI-T  (Mean±SD) | F | df | P |
| --- | --- | --- | --- | --- | --- | --- | --- | --- | --- |
| **Total** | 759 | 40.4±9.2 |  |  |  | 41.3±9.2 |  |  |  |
| **Age(Y)** |  |  | 0.925 | 2 | 0.397 |  | 1.722 | 2 | 0.179 |
| 18-25 | 24 | 40.7±8.3 |  |  |  | 42.6±9.2 |  |  |  |
| 26-39 | 549 | 40.7±9.7 |  |  |  | 41.9±9.5 |  |  |  |
| 40-59 | 186 | 39.6±8.0 |  |  |  | 40.3±8.3 |  |  |  |
| **Income** |  |  | 1.327 | 2 | 0.266 |  | 2.193 | 2 | 0.112 |
| Low | 347 | 41.0±9.5 |  |  |  | 42.1±9.6 |  |  |  |
| Middle | 256 | 40.0±9.1 |  |  |  | 41.0±9.0 |  |  |  |
| High | 156 | 39.8±8.8 |  |  |  | 40.3±8.6 |  |  |  |
| **Education** |  |  | 0.920 | 2 | 0.399 |  | 1.575 | 2 | 0.208 |
| High school or below | 260 | 41.0±9.1 |  |  |  | 42.0±9.3 |  |  |  |
| College or Bachelor | 436 | 40.2±9.5 |  |  |  | 41.2±9.2 |  |  |  |
| Master or Doctor | 63 | 40.4±9.2 |  |  |  | 39.8±8.6 |  |  |  |
| **Occupation** |  |  | 0.227 | 757 | 0.829 |  | 0.017 | 757 | 0.692 |
| Employees of institutions or government | 307 | 40.3±9.1 |  |  |  | 41.2±9.3 |  |  |  |
| Other employees or retired or students | 452 | 40.5±9.3 |  |  |  | 41.5±9.2 |  |  |  |

**Supplemental Table 1 Demographic characteristics of the included population**

Note. †, Data in this table were also presented in Figure 1 and 2. STAI-S, State-Trait Anxiety Inventory-State; STAI-T, State-Trait Anxiety Inventory-Trait; QG, quarantined group; Non-QG, non-quarantined group; Y, year; OI, Ovulation induction; IVF, In vitro fertilization; P values <0.05 are in bold typeface.
